# Adrenal Abscesses: A Systematic Review of the Literature

**DOI:** 10.3390/jcm12144601

**Published:** 2023-07-11

**Authors:** Nikola Gligorijevic, Marija Kaljevic, Natasa Radovanovic, Filip Jovanovic, Bojan Joksimovic, Sandra Singh, Igor Dumic

**Affiliations:** 1 Division of General Internal Medicine, Department of Medicine, University of Pittsburgh, Pittsburgh, PA 15213, USA; 2 Department of Hospital Medicine, Saint Francis Hospital and Medical Center, Hartford, CT 06105, USA; m.kaljevic@yahoo.com; 3 Division of Internal Medicine, University of Connecticut, Farmington, CT 06030, USA; 4 Department of Endocrinology, Dartmouth-Hitchcock Medical Center, Geisel School of Medicine, Dartmouth College, Hanover, NH 03755, USA; natasa.stankovic.md@gmail.com; 5 Department of Internal Medicine, Merit Health Wesley, Hattiesburg, MS 39402, USA; drfilipjovanovic91@gmail.com; 6 Department of Pathological Physiology, Faculty of Medicine Foca, University of East Sarajevo, 73300 Foca, Bosnia and Herzegovina; bojannjoksimovic@gmail.com; 7 Clinic for Endocrinology, Diabetes and Metabolic Diseases, Faculty of Medicine, University of Belgrade, 11000 Belgrade, Serbia; bastet224@yahoo.com; 8 Department of Hospital Medicine, Mayo Clinic Health System, Eau Claire, WI 54703, USA; igordumic84@gmail.com; 9 Mayo Clinic College of Medicine and Science, Rochester, MN 55905, USA

**Keywords:** adrenal abscess, adrenal infection, adrenal insufficiency, histoplasmosis

## Abstract

**Objective:** To summarize the existing knowledge about adrenal gland abscesses, including etiology, clinical presentation, common laboratory and imaging findings, management and overall morbidity and mortality. **Design:** Systematic literature review. **Methods:** We performed a search in the PubMed database using search terms: ‘abscess and adrenal glands’, ‘adrenalitis’, ‘infection and adrenal gland’, ‘adrenal abscess’, ‘adrenal infection’ and ‘infectious adrenalitis’. Articles from 2017 to 2022 were included. We found total of 116 articles, and after applying exclusion criteria, data from 73 articles was included in the final statistical analysis. **Results:** Of 84 patients included in this review, 68 were male (81%), with a mean age of 55 years (range: 29 to 85 years). Weight loss was the most frequent symptom reported in 58.3% patients, followed by fever in 49%. Mean duration of symptoms was 4.5 months. The most common laboratory findings were low cortisol (51.9%), elevated ACTH (43.2%), hyponatremia (88.2%) and anemia (83.3%). Adrenal cultures were positive in 86.4% cases, with *Histoplasma capsulatum* (37.3%) being the leading causative agent. Blood cultures were positive in 30% of patients. The majority of the adrenal infections occurred through secondary dissemination from other infectious foci and abscesses were more commonly bilateral (70%). A total of 46.4% of patients developed long-term adrenal insufficiency requiring treatment. Abscess drainage was performed in 7 patients (8.3%) and adrenalectomy was performed in 18 (21.4%) patients. The survival rate was 92.9%. Multivariate analysis showed that the only independent risk factor for mortality was thrombocytopenia (*p* = 0.048). **Conclusion:** Our review shows that adrenal abscesses are usually caused by fungal pathogens, and among these, *Histoplasma capsulatum* is the most common. The adrenal glands are usually involved in a bilateral fashion and become infected through dissemination from other primary sources of infection. Long-term adrenal insufficiency develops in 46% of patients, which is more common than what is observed in non-infectious etiology of adrenal gland disorders. Mortality is about 7%, and the presence of thrombocytopenia is associated with worse prognosis. Further prospective studies are needed to better characterize optimal testing and treatment duration in patients with this relatively rare but challenging disorder.

## 1. Introduction

Adrenal glands are retroperitoneal organs residing on the upper poles of the kidneys, acting as invaluable regulators of metabolic and stress responses. These glands are composed of two distinct parts: the outer (cortex) and inner (medulla) [1]. The adrenal cortex comprises three zones: zona glomerulosa, zona fasciculata, and zona reticularis, where synthesis of aldosterone, cortisol, and androgen hormones occurs, respectively [1,2]. The adrenal medulla is a neuroendocrine ganglion comprised of chromaffin cells that secrete catecholamines (epinephrine and norepinephrine) [3,4]. Disorders of the adrenal glands may be broadly classified as congenital or acquired, and as non-neoplastic or neoplastic. Non-neoplastic diseases of the adrenal gland are further divided into congenital, traumatic, infectious, hemorrhagic, hyperplastic, and adrenal cyst not associated with malignancy [5].

Chronic adrenal cortical insufficiency, or Addison’s disease, occurs when 90% or more of the adrenal cortex has been destructed. In developed countries, the most common cause of primary adrenal insufficiency is autoimmune adrenalitis [6], while in developing parts of the world, tuberculosis (TB) is the leading infectious cause [7,8]. In elderly and immunocompromised patients, opportunistic fungal infections such as histoplasmosis, paracoccidioidomycosis, and blastomycosis are particularly important causes [9]. These infections have been documented in numerous case reports to induce adrenal gland damage through abscess formation [10,11,12]. 

An abscess is an enclosed collection of pus in an organ or in the space between organs [13]. The clinical characteristics and epidemiology of abscesses in various organs have already been well described [14,15,16]. However, there is a paucity of literature showing the characteristics and outcome of abscesses in endocrine glands. Because adrenal gland abscesses are rare and pose significant clinical challenges, the aim of this review is to synthesize the available data on abscesses of adrenal glands by performing a systematic literature review on this topic. We analyzed the data regarding causative pathogens, clinical presentation, diagnosis, treatment, morbidity, and mortality. 

## 2. Materials and Methods

We performed a systematic review of the literature following Preferred Reporting Items for Systematic Review and Meta-Analysis (PRISMA) guidelines by searching the PubMed database for articles describing adrenal infections. We used the following search terms: ‘abscess and adrenal glands’, ‘adrenalitis’, ‘infection and adrenal gland’, ‘adrenal abscess’, ‘adrenal infection’ and ‘infectious adrenalitis’. All case reports and case series dating from January 2017 to July 2022 were included.

**Figure 1:**
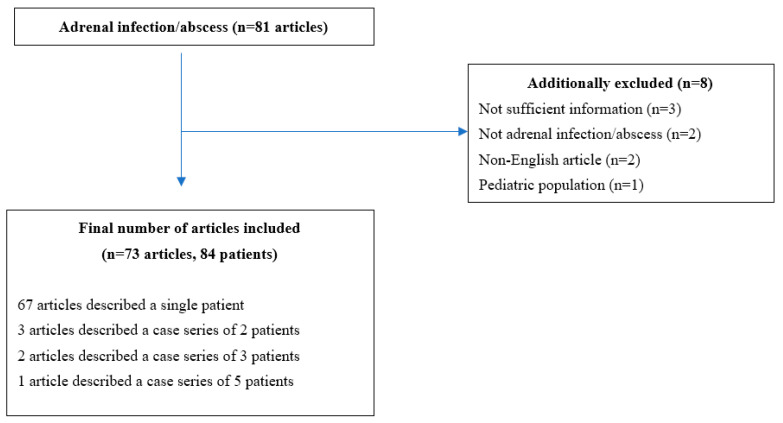
Flowchart of the literature search and article selection according to PRISMA guidelines.

Two authors (M.K. and N.G.) independently and blindly selected the cases and reached the final consensus after comparing their selections. All the cases with adrenal abscess in adults were included. Case reports not providing adequate amount of data, articles written in languages other than English and articles involving animal subjects were excluded from the analyses. The final number of articles included was 73, which resulted in a total of 84 patients [11,12,17,18,19,20,21,22,23,24,25,26,27,28,29,30,31,32,33,34,35,36,37,38,39,40,41,42,43,44,45,46,47,48,49,50,51,52,53,54,55,56,57,58,59,60,61,62,63,64,65,66,67,68,69,70,71,72,73,74,75,76,77,78,79,80,81,82,83,84,85,86,87] (Figure 1).

After the final selection of articles, we created an Excel table to enlist variables that included demographics (age, sex, race, ethnicity, body mass index, ethanol use, intravenous drug use, tobacco use history), past medical history (immunocompromised states, diabetes mellitus with complications, history of human immunodeficiency virus (HIV) infection, presence of malignancy currently on chemotherapy, autoimmune disease, prior history of adrenal dysfunction), presenting symptoms (fever, chills, anorexia, nausea, vomiting, skin discoloration, malaise, abdominal and back pain), duration of symptoms, presence of sepsis (sepsis was defined as presence of infection and at least two positive systemic inflammatory response syndrome (SIRS) criteria), common laboratory values and imaging modalities, tissue biopsy, type of adrenal involvement (primary vs. secondary, focal vs. disseminated, unilateral vs. bilateral), treatment (choice and duration) and outcome (if survived, then further classified by adrenal insufficiency vs. intact adrenal function). 

The nonparametric Fisher and Chi-square test and parametric *t*-test for independent samples were used to compare differences between groups for the univariate analysis of risk factors associated with adrenal insufficiency. A binary logistic regression model was used for the multivariate analysis to assess the association between mortality in patients with adrenal insufficiency and the multiple variables. All statistical analyses were performed using IBM SPSS Statistics Software version 24.0 for Windows (IBM Corp., Armonk, NY, USA). All *p*-values lower than 0.05 were considered statistically significant.

## 3. Results

We included 84 patients, of which 68 were male (81%), with a mean age of 55 years (range: 29 to 85 years). There was no statistically significant difference between the two groups (survivors and those who died) in comorbidities, immunosuppression, signs and symptoms, laboratory and culture findings, or age and genders (*p* > 0.05). Weight loss was the most frequent symptom (n = 49, 58.3%), followed by fever (n = 41; 48.8%), with a mean duration of symptoms of 4.51 months (range: 1 to 24 months).

Sepsis was present in 12 patients (14.3%). The most common laboratory findings were low cortisol levels (n = 42/81; 51.9%), high ACTH levels (n = 32/74, 43.2%), hyponatremia (n = 30/34, 88.2%) and anemia (n = 20/24, 83.3%). Blood cultures were positive in 10 of 33 patients (30.3%). The most common pathogen isolated from blood was *Nocardia* spp. (n = 3/10, 30%). Adrenal cultures were positive in 51/59 patients (86.4%). The most common isolate from adrenal biopsies was *Histoplasma capsulatum* (n = 19/51; 37.3%), followed by *Mycobacterium tuberculosis* in 10/51 patients (19.6%), *Paracoccidioides* spp. in 5/51 patients (9.8%) and *Cryptococcus* spp. in 4/51 patients (7.8%). Adrenal glands were usually affected secondarily (n = 40, 47.6%) in patients with disseminated infections (n = 53, 63.1%), and bilateral involvement is more common (n = 59, 70.2%).

Diagnosis was most commonly established by abdominal ultrasound (US), computed tomography (CT) and magnetic resonance imaging (MRI). Antibacterial therapy was used in 51 patients (60.7%), while antifungal therapy was used in 42 patients (50%), with a mean duration of targeted therapy of 161.18 days (range: 10 to 1095 days). The empirical therapy was accurate in 38 from 51 patients (74.5%), while hormonal replacement therapy during initial treatment was used in 47 patients (56%). Abscess drainage was performed in 7 patients (8.3%) and adrenalectomy was performed in 18 (21.4%) patients. A total of 39 (46.4%) patients developed long-term adrenal insufficiency which required treatment. The survival rate in our review was 92.9%, and six patients died (7.1%). Univariate analysis demonstrated that patients who died had a significantly higher frequency of chronic kidney disease (*p* = 0.006), a higher frequency of thrombocytopenia (*p* = 0.033) and a higher frequency of increased AST and ALT (*p* = 0.049). Survivors had a significantly higher frequency of anorexia (*p* = 0.041), a higher frequency of low cortisol levels (*p* = 0.031) and higher frequency of hormone replacement necessary during the initial treatment (*p* = 0.044). 

Multivariate analysis showed that an independent risk factor for mortality was thrombocytopenia (*p* = 0.048). Our review found that patients who develop thrombocytopenia have a two-times-greater risk of dying than those who do not (OR = 2.175; *p* = 0.048).

## 4. Discussion

### 4.1. Demographics

As outlined in the results above, males were more affected than females. Gender and age do not seem to be a risk factor for the development of adrenal abscess, nor do they appear to be a risk factor for mortality in this group of patients. Adrenal involvement (either primary or as a part of disseminated disease) is more common among males, but the reasons for this tropism are not yet understood [88].

### 4.2. Symptoms

#### 4.2.1. Weight Loss

Weight loss was the most common symptom observed among the reviewed patients. It is well known that weight loss occurs frequently during any infectious process, predominantly due to anabolic losses and increased catabolic and basal metabolic rate. This is particularly true for subacute and chronic infections, such as those of the adrenal glands [89,90]. Concentration of ghrelin, the appetite hormone, increases when cortisol levels increase [91]. Similarly, with hypocortisolemia, the body produces less ghrelin, which in turn leads to poor or complete loss of appetite. Additionally, in adrenal crisis, there is no inhibition of corticotropin-releasing hormone (CRH) secretion, which further suppresses the appetite [92]. CRH is an appetite suppressant—it inhibits neuropeptide Y, which is known to stimulate hunger [93,94]. Adrenal exhaustion is a term used when cortisol levels drop excessively. This might lead to hypoglycemia, which can further cause weight loss. In addition, interleukin-1 is a very potent cytokine with anorexic effects, leading to reduction of food intake and weight loss in febrile illnesses [95]. Lack of cortisol inhibition on production of thyroid-stimulating hormone (TSH) leads to a hypermetabolic hyperthyroid state [92]. The interplay of all the above-mentioned mechanisms might be the reason for observed appetite suppression, anorexia, and weight loss in patients with adrenal abscesses and adrenal insufficiency (AI). 

#### 4.2.2. Fever

Almost half of the patients in the review presented with a fever. Exogenous pyrogens (such as bacteria, viruses and fungi) can induce fever by activating host cells to release interleukins. Endogenous pyrogens (proinflammatory cytokines such as interleukin-1 (IL-1), interleukin-6 (IL-6), tumor necrosis factor (TNF) and interferon) elevate the body temperature by acting on the hypothalamus directly, through induction of prostaglandin synthesis in organum vasculosum, raising the temperature set-point and initiating a febrile response [95]. AI itself can present with persistent fever, even in the absence of an infectious pathogen [96]. Fever and elevated inflammatory markers cause an increase in inflammatory cytokine production, which further leads to a rise in endogenous pyrogen production [97]. C reactive protein (CRP) is produced by hepatocytes and regulated by IL-6 and IL-1b, and it is believed that AI can trigger these processes, but these mechanisms have yet to be determined [97]. Fever increases basal metabolic rate by 10–13% for each Celsius degree rise [90,98]. We suggest that most of the patients in our review developed fever likely due to the effect of exogenous pyrogens, such as microorganisms. In the cases with concurrent AI, this could be a contributory mechanism, and fever indeed is a multifactorial phenomenon. 

#### 4.2.3. Hyperpigmentation

Approximately one third of the patients developed skin hyperpigmentation. Destruction of the adrenal gland caused by an infection can lead to primary adrenal insufficiency and adrenocorticotropic hormone elevation (ACTH) due to closed-loop regulation of the HPA axis [99]. Elevation in ACTH causes the activation of melanocortin-1 receptors (M-1R), responsible for pigmentation, resulting in mucocutaneous hyperpigmentation [100]. Changes are mostly visible on sun-exposed areas (knuckles, elbows, knees), but buccal membranes and skin around the lips can be affected as well.

#### 4.2.4. Abdominal Pain

Adrenal glands are richly innervated by the celiac plexus, which provides sensory signals about pathological processes within and around the adrenal glands [101]. Connective tissue forms a capsule that surrounds the adrenal glands [102]. As the abscess develops, the adrenal capsule may become distended due to purulent material accumulation within the gland and its capsule, which further leads to painful sensations.

### 4.3. Laboratory Markers

#### 4.3.1. Anemia

Anemia in patients with adrenal infections is anemia of chronic disease due to protracted infection and chronic ongoing inflammation. This subtype of anemia occurs due to functional iron deficiency caused by different pathophysiological mechanisms, such as excess hepcidin production, suppressed erythropoietin production and reduced lifespan of red blood cells [103,104,105,106,107]. Additionally, histoplasma might infiltrate bone marrow and contribute to the development of anemia (and pancytopenia) through this mechanism. 

#### 4.3.2. Thrombocytopenia and Sepsis

Thus far, no study has examined the rate of sepsis development in patients with adrenal abscesses. Similarly, it has not been previously demonstrated that the presence of thrombocytopenia in these patients portends worse prognosis. In this review, we found that approximately 12 out of 84 total patients developed sepsis as a complication of adrenal abscess. Adrenal infectious are infrequently implicated as a source of sepsis and this remains a rare etiology, unlike more common diagnoses such as pneumonia, skin and soft tissue infection and genitourinary infections [108]. In patients with adrenal abscess, sepsis might occur due to the infection spreading from the primary infected adrenal gland, or the adrenal gland might be infected secondarily during sepsis, from bacteremia seeding or from other sources. In 27 patients, out of 29 where the primary source of infection was reported, the primary source of infection was in another organ, and the adrenal gland was secondarily affected.

Thrombocytopenia is defined as an absolute platelet count below 150/μL [109]. The causes of thrombocytopenia are various and infectious causes are among the most common [110]. Mechanisms of thrombocytopenia during infection, including its most severe form sepsis, include bone marrow suppression, platelet destruction, altered thrombopoiesis, hemodilution, coagulopathy and endothelial dysfunction. Thrombocytopenia is a marker of organ dysfunction and has predictive value for severity of sepsis and outcome [111,112,113]. 

Thrombocytopenia has been investigated in many infectious diseases as a prognostic factor for disease severity and the outcome. One prospective study documented that more than 55% of patients with sepsis develop thrombocytopenia, and that patients who also have bacteremia and extrapulmonary infection are more likely to develop thrombocytopenia [111]. One study from Sweden investigated the rate of thrombocytopenia in patients with bacteremia. They found that about 20–30% of patients with bacteremia develop thrombocytopenia, and rates were higher for patients with *Methicillin-resistant Staphylococcus aureus (MRSA)* bacteremia when compared to those with *E. coli* or *Streptococcus* pneumonia [114].

We found that thrombocytopenia was a predictor for mortality, which correlates with the findings of the above studies. Hence, the platelet count might be a useful hematological marker to follow in patients with adrenal abscess during the acute phase of the illness to inform the risk of mortality.

#### 4.3.3. Hyponatremia

Given the high percentage of individuals with a proven AI, we assume that this is the predominant underlying pathophysiology of hyponatremia in this patient population. Hyponatremia occurs in both primary and secondary adrenal insufficiency, but the mechanism in these two entities is different [115]. Low cortisol level in secondary adrenal insufficiency leads to an increase in vasopressin release through pituitary stimulation and vasoplegia [116], hence causing free-water retention and subsequent hyponatremia. In Addison’s disease, in addition to cortisol deficiency, aldosterone deficiency plays an important role in development of hyponatremia, predominantly through renal sodium wasting and hypovolemia [115,116,117,118].

#### 4.3.4. Adrenal Insufficiency (AI)

AI is characterized by glucocorticoid deficiency and the most common symptoms associated with it include fatigue, weakness, abdominal pain, hypotension, weight loss and generalized skin hyperpigmentation associated with primary adrenal insufficiency [119]. Low cortisol level and elevated ACTH are laboratory hallmarks of the disease, while hypoglycemia, hyponatremia and hyperkalemia are also frequently identified. In our review, 51.9% of patients (42/81) had a low cortisol level and 43.2% (32/74) had an elevated level of ACTH. The etiology of AI can be infectious and noninfectious. Among noninfectious etiologies, autoimmune adrenalitis, hemorrhage and malignancy are the most common ones [120,121,122,123,124].

Due to their similarity in symptoms with AI, adrenal infections are oftentimes an overlooked and patient outcomes can be severely affected by a delay in diagnosis and treatment [125]. The most common infectious agents include *Mycobacterium tuberculosis*, *Mycobacterium Avium Complex*, Meningococcus, *Histoplasma capsulatum*, *Blastomyces dermatoidides*, *Paracoccidiodes brasiliensis*, *Cryptococcus neoformans* and *Coccicioides immitis*. Our review indicates that 46.4% of patients with adrenal infections developed long-term adrenal insufficiency. We conclude that local destruction of large portions of the adrenal glands is most likely the mechanism responsible for the high percentage of AI in infected glands.

### 4.4. Adrenal Histopathology

In 86.4% of the cases, a causative pathogen was isolated, either using adrenal gland fine-needle aspiration, abscess drainage or microbiologic tissue evaluation following adrenalectomy.

**Figure 2:**
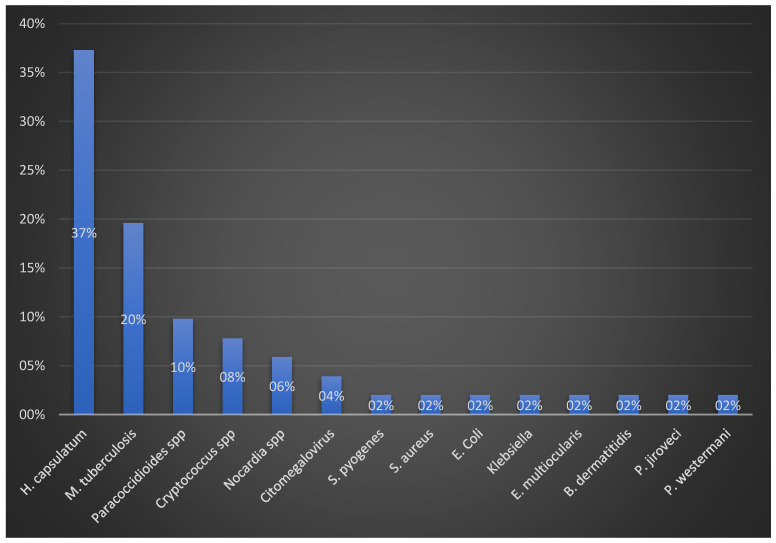
Isolated pathogens in adrenal biopsy samples.

In this review, fungal etiology was predominant, with *Histoplasma capsulatum* being the leading cause (Figure 2). The high rate of adrenal infections caused by *Histoplasma capsulatum* is not a surprise, given the tropism of the fungi for the adrenal gland [126]. However, this finding somewhat differs from the previous studies indicating TB as the major infectious etiology of Addison’s disease, especially in developing countries [127]. The reasons for changes in this trend are multiple, including global decrease in TB adrenalitis incidence in developed countries [121] and an increase in diagnosing and reporting of histoplasmosis cases worldwide [128]. *Histoplasma capsulatum* is found in damp soil rich in bird and bat excreta [129]. Recent publications suggest that climate change and higher land utilization by humans may alter local conditions, leading to greater exposure to *Histoplasma* and its more rapid spread [129]. Furthermore, the increase in global travel, migration, and percentage of immunocompromised population over the last decade are likely contributing to the observed higher incidence of this infection [130]. In the United States, this is also reflected through the detected rise in histoplasmosis-related hospitalizations since the beginning of the century [131]. Overall, the findings of our review align with these trends.

Among bacterial etiologies, *Mycobacterium tuberculosis* was found to be the most common, causing 20% of the cases with a positive adrenal cultures.

We observed differences in the frequency of certain infectious etiologies between adrenal and other endocrine gland abscesses. A recently published multicenter study on pituitary abscesses showed that Gram-positive bacteria, such as *Staphylococcus* spp. and *Streptococcus* spp., were among the most common causes, with a low incidence of fungal agents [132]. Similarly, suppurative thyroiditis is predominantly caused by bacteria [133], and according to a recent systematic review on thyroid abscesses, bacterial etiology is found in approximately 70–80% of cases [134,135,136,137,138,139]. Interestingly, we detected only a small number of cases that were caused by bacterial agents other than *Mycobacterium tuberculosis*, including Gram-negative rods (*E. coli*, *Klebsiella pneumoniae*), *Nocardia* spp. and Gram-positive cocci (*Streptococcus pyogenes*, *Staphylococcus aureus*). In this review, we did not find any correlation between specific pathogens and mortality.

### 4.5. Type of Adrenal Involvement

Infectious agents can affect adrenal glands in a several different ways. Direct damage to adrenal glands occurs when microbial agents replicate and endotoxins and/or exotoxins are produced within the gland. Indirect damage occurs when there is a dysregulation between immunologic and endocrine signaling in response to damage caused by a microbial agent at a distant site [126]. 

**Figure 3:**
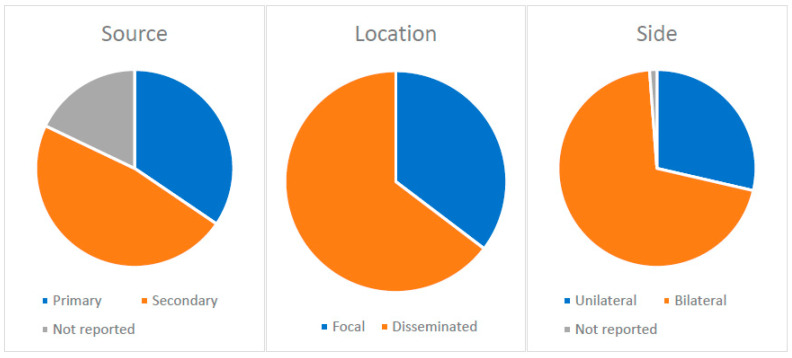
Adrenal involvement: source, location and side.

Adrenal glands are susceptible to infections (either primary or secondary by dissemination from other infectious focus). The explanation likely lies in HPA axis activation and excess cortisol production as a stress response to infection anywhere in the body. Hypercortisolemia induced by stress causes T-cell-mediated immunity alterations on the level of the adrenal glands [126,140] that leads to increased susceptibility to infections. It is known that endotoxins excreted by bacteria can cause prolonged activation of the HPA axis due to release of IL-1, IL-6 and TNF-α from peripheral immune cells, acting directly on corticotropin-releasing factor [126,140]. Suppression of cell-mediated local immunity due to the presence of cortisol within the adrenal gland and lack of reticuloendothelial cells within glands represent the desired milieu for the uninhibited growth of fungi. This also supports the explanation for the above-mentioned tropism of histoplasma for the adrenal gland [141]. Details regarding the characteristics of adrenal involvement in our review are depicted in Figure 3.

The adrenal gland is richly vascularized, which could explain the frequent hematogenous dissemination from different primary sites of infection [142,143]. When it comes to Mycobacterium specifically, the cortex is usually involved, but the medulla can also be affected [144].

### 4.6. Imaging Findings

CT scanning remains the first-level imaging modality for the evaluation of the adrenal glands [145]. In 88.1% of patients in our review, a CT scan was performed. Positron emission tomography (PET) scan and MRI were used less frequently, in 22.6 and 17.9% of cases, respectively. The significant increase in the utilization of CT scanning over the last three decades [146], its wider accessibility and relatively lower cost compared to other imaging modalities, are likely the reasons behind these results.

The most common CT findings were bilateral diffuse adrenal enlargement, heterogeneously enhancing adrenal lesions, nodular changes and diffuse adrenal thickening with peripheral enhancement. An advantage of using the CT scan in the cases of suspected adrenal involvement was detecting potential intrabdominal and intrathoracic infection dissemination without the need to utilize additional imaging studies.

### 4.7. Therapy

**Figure 4:**
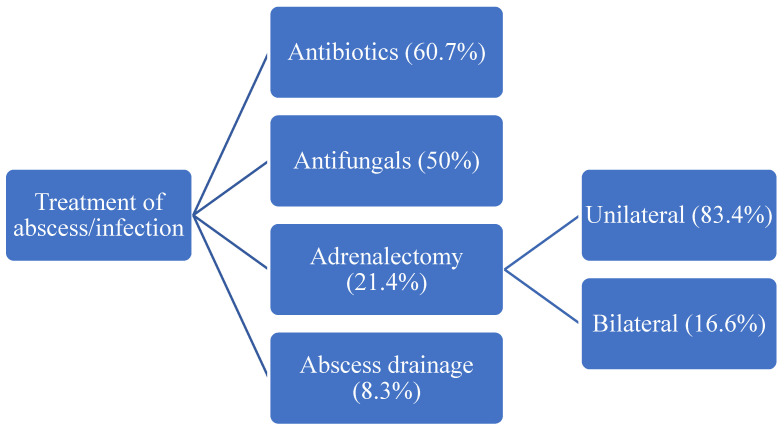
Treatment modalities used in adrenal abscesses.

The mainstay of adrenal abscess treatment is the use of antimicrobial therapy, with or without abscess drainage. In many instances, empiric broad-spectrum antibiotics are initiated in the early stages of the disease, but later modified, based on the culture and sensitivity data (Figure 4).

Our review indicates that the majority of cases, in fact, had an adequate empirical therapy prior to final culture reports. We believe that when patients present with signs and symptoms of adrenal disease in endemic regions, certain pathogens, such as fungi, are suspected almost immediately, and hence adequate empirical antimicrobial treatment is initiated early in the course of illness. It is of extreme importance to obtain blood cultures, fungal markers and histopathologic tissue analysis (when possible) for diagnosis confirmation, so that catastrophic consequences and complications can be avoided.

Early antifungal therapy can lead to partial recovery of the adrenal gland even though most cases require long-term corticosteroid maintenance therapy. Furthermore, early antifungal therapy is thought to prevent fungal embolic events, which further leads to reduced adrenal necrosis and prevents progression to adrenal dysfunction [125,147].

The duration of treatment of adrenal infections is prolonged in the majority of cases, likely due to the nature of the isolated pathogens, which require prolonged treatment in general, to ensure cure and reduction of recurrence [148].

**Figure 5:**
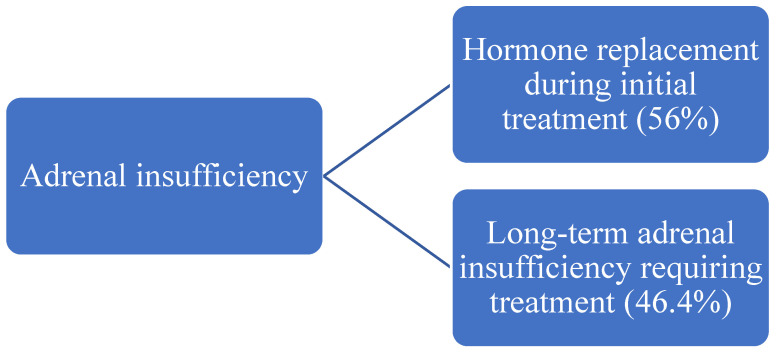
Initial and long-term adrenal insufficiency.

When it comes to long-term hormone replacement therapy, the data of our review show that up 46.4% of patients with adrenal infection depend on lifelong corticosteroid therapy (Figure 5).

## 5. Limitations of the Study

This review has a few limitations. First, we evaluated cases from only one database, and we have included only case reports that were written in English. By doing this, we might have inadvertently missed some high-quality reports. The second limitation, which is universal for this type of review, is the possibility of selection bias ( not all cases are reported, and patient with extreme outcomes might have be overrepresented). It is likely that not all cases of adrenal abscesses have been reported and the ones with unusual pathogens or unexpected outcomes are more likely to be reported. Finally, we have not described viral adrenalitis in this review, since our focus was on the abscesses and since it has been described already elsewhere [149].

## 6. Conclusions

Few studies have reported infections of the adrenal glands thus far [126,149]. Hence, this is a novel report that describes all aspects of adrenal abscesses based on the currently available literature. In summary, adrenal infections are usually caused by fungal pathogens, and among these *Histoplasma capsulatum* is the most common. AI develops in 46% of patients, which is more common than what is observed in non-infectious etiology of adrenal gland disorders. The majority of patients present with fever and weight loss, which is multifactorial and related to both infection per se and AI. Adrenal abscesses are rarely the primary focus of infection, and most commonly the glands are infected through hematogenous dissemination from another organ. Targeted antimicrobial therapy is a mainstay of treatment, while in about 20% of cases adrenalectomy is needed for source control. Mortality is about 7%. Further prospective studies are needed to better characterize optimal testing and treatment duration in patients with this relatively rare but challenging disorder. 

## Data Availability

All data are publically available on PubMed and from corresponding author upon request.

## References

[1] Pignatti E., Leng S., Carlone D.L., Breault D.T. (2017). Regulation of zonation and homeostasis in the adrenal cortex. Mol. Cell. Endocrinol..

[2] Srinivasan S., Shariff M., Bartlett S.E. (2013). The role of the glucocorticoids in developing resilience to stress and addiction. Front. Psychiatry.

[3] Carbone E., Borges R., Eiden L.E., García A.G., Hernández-Cruz A. (2019). Chromaffin Cells of the Adrenal Medulla: Physiology, Pharmacology, and Disease. Compr. Physiol..

[4] Burford N.G., Webster N.A., Cruz-Topete D. (2017). Hypothalamic-Pituitary-Adrenal Axis Modulation of Glucocorticoids in the Cardiovascular System. Int. J. Mol. Sci..

[5] Turgut A.O.M., Akpinar V., MacLennan D., MacLennan G., Dogra V.M.G. (2013). Congenital and Acquired Nonneoplastic Adrenal Diseases. Genitourinary Radiology: Male Genital Tract, Adrenal and Retroperitoneum.

[6] Michels A.W., Eisenbarth G.S. (2010). Immunologic endocrine disorders. J. Allergy Clin. Immunol..

[7] Azeez T.A., Irojah O.A., Lakoh S., Lawal A., Ajiboso O. (2021). A systematic review of adrenal insufficiency among patients with pulmonary tuberculosis in Sub-Saharan Africa. Int. J. Mycobacteriol..

[8] Gupta S., Ansari M.A.M., Gupta A.K., Chaudhary P., Bansal L.K. (2022). Current Approach for Diagnosis and Treatment of Adrenal Tuberculosis-Our Experience and Review of Literature. Surg. J..

[9] Upadhyay J., Sudhindra P., Abraham G., Trivedi N. (2014). Tuberculosis of the adrenal gland: A case report and review of the literature of infections of the adrenal gland. Int. J. Endocrinol..

[10] Kathuria S., Capoor M.R., Yadav S., Singh A., Ramesh V. (2013). Disseminated histoplasmosis in an apparently immunocompetent individual from north India: A case report and review. Med. Mycol..

[11] Cabrera N.L., Malek A.E., Shelburne S., Taremi M., Awadh H., Francisco D., Robins A., Jabbour E., Chemaly R.F. (2020). Disseminated cytomegalovirus infection with bilateral adrenal pseudotumors masquerading as recurrent hematologic malignancy. Infection.

[12] Jin W., Miao Q., Wang M., Zhang Y., Ma Y., Huang Y., Wu H., Lin Y., Hu B., Pan J. (2020). A rare case of adrenal gland abscess due to anaerobes detected by metagenomic next-generation sequencing. Ann. Transl. Med..

[13] Singer A.J., Talan D.A. (2014). Management of skin abscesses in the era of methicillin-resistant Staphylococcus aureus. N. Engl. J. Med..

[14] Brouwer M.C., Coutinho J.M., van de Beek D. (2014). Clinical characteristics and outcome of brain abscess: Systematic review and meta-analysis. Neurology.

[15] Losie J.A., Lam J.C., Gregson D.B., Parkins M.D. (2021). Epidemiology and risk factors for pyogenic liver abscess in the Calgary Health Zone revisited: A population-based study. BMC Infect. Dis..

[16] Falhammar H., Wallin G., Calissendorff J. (2019). Acute suppurative thyroiditis with thyroid abscess in adults: Clinical presentation, treatment and outcomes. BMC Endocr. Disord..

[17] Grover S., Selhi P.K., Sood N., Sood R., Kaur H., Mbbs R.S. (2017). “Polka Dot Macrophages” on cytology of bilateral adrenal masses-Nailing disseminated histoplasmosis. Diagn. Cytopathol..

[18] Grüter B.E., Reuss A.M., Rushing E.J., Pangalu A., Oertel M.F. (2021). An unexpected intracerebral lesion—Case report of a superinfected aspergillosis mimicking a brain metastasis. BMC Infect. Dis..

[19] Robinson L.J., Lu M., Elsayed S., Joy T.R. (2019). Bilateral adrenal histoplasmosis manifesting as primary adrenal insufficiency. CMAJ.

[20] Tran N.Q., Phan C.C., Doan T.T.P., Tran T.V. (2021). Bilateral adrenal masses due to tuberculosis: How to diagnose without extra-adrenal tuberculosis. Endocrinol. Diabetes Metab. Case Rep..

[21] Koh S.A. (2018). Addison’s disease due to bilateral adrenal tuberculosis on 18F-fluorodeoxyglucose positron emission tomography computed tomography. Infect. Dis. Rep..

[22] Yang N., Zhou L., Mo X., Huang G., Wu P. (2021). Successful treatment of severe electrolyte imbalance-induced cardiac arrest caused by adrenal tuberculosis with ECMO in the ED. Int. J. Emerg. Med..

[23] Chee D., Moritz A.W., Profit A.P., Agarwal A.N., Anstead G.M. (2021). Fatal coccidioidomycosis involving the lungs, brain, tongue, and adrenals in a cirrhotic patient. An autopsy case. IDCases.

[24] Roxas M.C.A., Sandoval M.A.S., Salamat M.S., Matias P.J., Cabal N.P., Bartolo S.S. (2020). Bilateral adrenal histoplasmosis presenting as adrenal insufficiency in an immunocompetent host in the Philippines. BMJ Case Rep..

[25] Tee S.A., Gan E.H., Kanaan M.Z., Price D.A., Hoare T., Pearce S. (2018). An unusual cause of adrenal insufficiency and bilateral adrenal masses. Endocrinol. Diabetes Metab. Case Rep..

[26] Lieu A., Church D., Vaughan S. (2021). Bilateral Adrenal Histoplasmosis in an Immunocompetent Host. Am. J. Trop. Med. Hyg..

[27] Kurian M.E., Jebasingh F.K., Kodiatte T.A., Thomas N. (2021). Adrenal histoplasmosis: An uncommon presentation with an ulcer of the tongue. BMJ Case Rep..

[28] Ramesh V., Narreddy S., Gowrishankar S., Barigala R., Nanda S. (2021). A challenging case of pyrexia of unknown origin: Adrenal histoplasmosis mimicking tuberculosis in a patient with chronic hepatitis C. Trop. Dr..

[29] Zhao N., Gao Y., Ni C., Zhang D., Zhao X., Li Y., Sun B. (2021). An autopsy case of unexpected death due to Addison’s disease caused by adrenal tuberculosis. Eur. J. Med. Res..

[30] Ito M., Hinata T., Tamura K., Koga A., Ito T., Fujii H., Hirata F., Sakuta H. (2017). Disseminated Cryptococcosis with Adrenal Insufficiency and Meningitis in an Immunocompetent Individual. Intern. Med..

[31] Peçanha-Pietrobom P.M., Falqueto A., Rodrigues Gandarella A.D., Moyzés J.V., Rangel K.A., Miranda L.B., Hemerly M.C., Careta R.S., Peçanha P.M. (2019). Case Report: Paracoccidioidomycosis in Solid Organ Transplantation: Disseminated Disease in a Liver Recipient and Literature Review. Am. J. Trop. Med. Hyg..

[32] Liu H., Tang T.J., An Z.M., Yu Y.R. (2022). Unilateral adrenal tuberculosis whose computed tomography imaging characteristics mimic a malignant tumor: A case report. World J. Clin. Cases.

[33] Soedarso M.A., Nugroho K.H., Meira Dewi K.A. (2018). A case report: Addison disease caused by adrenal tuberculosis. Urol. Case Rep..

[34] van Haren Noman S., Visser H., Muller A.F., Limonard G.J. (2018). Addison’s Disease Caused by Tuberculosis: Diagnostic and Therapeutic Difficulties. Eur. J. Case Rep. Intern. Med..

[35] Herreros B., Plaza I., García R., Chichón M., Guerrero C., Pintor E. (2018). Miliary Tuberculosis Presenting with Hyponatremia and ARDS in an 82-Year-Old Immunocompetent Female. Pathogens.

[36] Govind P., Subramanian K., Kumar S. (2022). Diagnostic and Therapeutic Implications of Organic Delusional Disorder due to Tuberculous Adrenalitis. Case Rep. Psychiatry.

[37] Maciel G.V.R., Tavares M.C.F., Pereira L.S., Silva G.L.C., De Oliveira N.R., Paulino E., Pascoal-Xavier M.A. (2018). Disseminated mycosis in a patient with yellow fever. Autops. Case Rep..

[38] Hatakeyama Y., Nakakubo S., Kusaka H., Watanabe N., Yoshida Y., Shinzaki H., Hiroumi H., Kishida N., Konno S. (2022). Listeria monocytogenes bacteremia mimicking the systemic metastasis of adrenal cancer: A case report. BMC Infect. Dis..

[39] Arambewela M., Ross R., Pirzada O., Balasubramanian S.P. (2019). Tuberculosis as a differential for bilateral adrenal masses in the UK. BMJ Case Rep..

[40] de Oliveira F.M., Fragoso M.C.B.V., Meneses A.F., Vilela L.A.P., Almeida M.Q., Palhares R.B., Mattos T.V.D.A., Scalissi N.M., Lima J.V. (2019). Adrenal insufficiency caused by paracoccidioidomycosis: Three case reports and review. AACE Clin. Case Rep..

[41] Qu F., Qu Z., Lv Y., Song B., Wu B. (2020). Disseminated Cryptococcosis revealed by transverse myelitis in Immunocompetent patient: A case report and review of the literature. BMC Neurol..

[42] Muhammed H., Nampoothiri R.V., Gaspar B.L., Jain S. (2018). Infectious causes of Addison’s disease: 1 organ-2 organisms!. BMJ Case Rep..

[43] Tam D.S., Man C.H., Wong K.W., Ng K.C. (2021). Emergency single-port laparoscopic partial adrenalectomy for adrenal abscess in an adult with disseminated *Streptococcus pyogenes* bacteraemia: A case report. Hong Kong Med. J..

[44] Agrawal J., Bansal N., Arora A. (2020). Disseminated histoplasmosis in India presenting as addisonian crisis with epiglottis involvement. IDCases.

[45] Majeed A., Kapoor V., Latif A., Zangeneh T. (2017). A 30-year delayed presentation of disseminated histoplasmosis in a heart transplant recipient: Diagnostic challenges in a non-endemic area. BMJ Case Rep..

[46] Gaspar G.G., Cocio T.A., Guioti-Puga F., Nascimento E., Fabro A.T., Kress M.R.V.Z., Bagagli E., Martinez R. (2020). Paracoccidioidomycosis due to Paracoccidioides lutzii complicated with adrenal injury and pulmonary arterial hypertension. Rev. Inst. Med. Trop. Sao Paulo.

[47] Mahajan V.K., Raina R.K., Singh S., Rashpa R.S., Sood A., Chauhan P.S., Mehta K.S., Rawat R., Sharma V. (2017). Case Report: Histoplasmosis in Himachal Pradesh (India): An Emerging Endemic Focus. Am. J. Trop. Med. Hyg..

[48] Jayathilake W.A.P.P., Kumarihamy K.W.M.P.P., Ralapanawa D.M.P.U.K., Jayalath W.A.T.A. (2020). A Rare Presentation of Possible Disseminated Histoplasmosis with Adrenal Insufficiency Leading to Adrenal Crisis in an Immunocompetent Adult: A Case Report. Case Rep. Med..

[49] Jackson C., McCullar B., Joglekar K., Seth A., Pokharna H. (2017). Disseminated Nocardia Farcinica Pneumonia with Left Adrenal Gland Abscess. Cureus.

[50] Xydakis A.M., Chatzellis E., Kolomodi D., Kaltsas G.A., Alexandraki K.I. (2020). Adrenal Failure and Orchitis Secondary to Tuberculosis Mimicking Metastatic Malignancy. Am. J. Med..

[51] Bender K., Waldie A.M., Asogan M., Figtree M.C., Sywak M.S. (2019). Fungal granuloma: A case report of a rare cause for isolated adrenal incidentaloma. ANZ J. Surg..

[52] Yu J., Lu Y., Han B. (2020). Primary adrenal insufficiency due to adrenal tuberculosis: A case report. J. Int. Med. Res..

[53] Wan S., Du F., Wang J., Bao J., Mi J., Sun X. (2021). Primary unilateral and epilepsy adrenal tuberculosis misdiagnosed as adrenal tumor: Report of two cases. Asian J. Surg..

[54] Ranawaka N., Welikumbura N.H. (2021). Addison’s disease as a primary manifestation of extrapulmonary tuberculosis; A case report. Indian J. Tuberc..

[55] Hasegawa M., Ito Y., Osugi Y., Hashimoto M., Hashimoto N., Yano K. (2022). Extrapulmonary pneumocystosis in an antiretroviral therapy-naïve, HIV-positive patient. Int. J. Infect. Dis..

[56] Kusuki K., Watanabe S., Mizuno Y. (2019). Tuberculous Addison’s disease with increased hydrocortisone requirements due to administration of rifampicin. BMJ Case Rep..

[57] Sharma B., Nehara H.R., Bhavi V.K., Maan P., Saran S. (2020). Adrenal histoplasmosis in immunocompetent individuals a case series from the North-Western part of India, Rajasthan province: An emerging endemic focus. Indian J. Med. Microbiol..

[58] Herndon J., Nadeau A.M., Davidge-Pitts C.J., Young W.F., Bancos I. (2018). Primary adrenal insufficiency due to bilateral infiltrative disease. Endocrine.

[59] Aziz H., Adam N.L., Karim N.A. (2021). Hypercalcaemia associated with disseminated cryptococcosis. BMJ Case Rep..

[60] Stankard M., Gopireddy D., Lall C. (2020). Role of MRI in the Diagnosis of Large Right Adrenal Abscess. Cureus.

[61] Kalinoski T. (2022). Waterhouse-Friderichsen Syndrome with Bilateral Adrenal Hemorrhage Associated with Methicillin-Resistant Staphylococcus aureus (MRSA) Bacteremia in an Adult Patient with History of Intravenous Drug Use. Am. J. Case Rep..

[62] Motta J.C., Barrera E.C. (2020). Acute adrenal insufficiency due to paracoccidiodomycosis. Report of 2 cases. Med. Mycol. Case Rep..

[63] Abdulla M.C. (2021). Reversible cerebellar ataxia and bipolar disorder secondary to tuberculous adrenalitis. Int. J. Mycobacteriol..

[64] Madhavan P., Nallu R., Luthra P. (2020). Histoplasmosis: An Unusual Cause of Adrenal Insufficiency. AACE Clin. Case Rep..

[65] Yaqoob H., Munawar M.M., Salih O., Deonarine A. (2021). Disseminated coccidioidomycosis in a patient who is immunocompromised in the setting of immune reconstitution inflammatory syndrome. BMJ Case Rep..

[66] Van Bogaert C., Vierasu I., Mathey C., Theunissen A., Goldman S. (2022). Bilateral cytomegalovirus infection of the adrenal glands revealed by ^18^F-FDG PET/CT in a patient with T-cell lymphoma. Clin. Case Rep..

[67] Agrawal S., Goyal A., Agarwal S., Khadgawat R. (2019). Hypercalcaemia, adrenal insufficiency and bilateral adrenal histoplasmosis in a middle-aged man: A diagnostic dilemma. BMJ Case Rep..

[68] Gaur M., Sethi J., Mitra S., Gupta K. (2021). Adrenal histoplasmosis presenting as life-threatening adrenal insufficiency. BMJ Case Rep..

[69] Šimeková K., Rosoľanka R., Szilágyová M., Antolová D., Nováková E., Novák M., Laca Ľ., Sadloňová J., Šoltys J. (2021). Alveolar Echinococcosis of the Liver with a Rare Infiltration of the Adrenal Gland. Helminthologia.

[70] Thijs E., Wierckx K., Vandecasteele S., Van den Bruel A. (2019). Adrenal insufficiency, be aware of drug interactions!. Endocrinol. Diabetes Metab. Case Rep..

[71] Li K., Ma Y., Ban R., Shi Q. (2021). Case Report: Diagnosis of Human Alveolar Echinococcosis via Next-Generation Sequencing Analysis. Front. Genet..

[72] Jiang H., Li A., Liao S., Ke S., Ji Z., Tian M., Zhang H. (2022). Simultaneous adrenal tuberculosis and renal oncocytoma mimicking malignant masses incidentally detected by ^18^F-FDG PET/CT in a patient with lymphoma. Eur. J. Nucl. Med. Mol. Imaging.

[73] Teng Q., Fan B., Wang Y., Wen S., Wang H., Liu T., Wang L. (2021). Primary adrenal tuberculosis infection in patients with Behcet’s disease presenting as isolated adrenal metastasis by ^18^F-FDG PET/CT: A rare case report and literature review. Gland Surg..

[74] Hsu J.L., Tjarks B.J., Berg A., Oliver T. (2017). Disseminated Blastomycosis Mimicking Malignancy. S. Dak. Med..

[75] Zhou J., Lv J., Pan Y., Xie J., Zhang Y. (2017). Unilateral Adrenal Cryptococcosis on FDG PET/CT. Clin. Nucl. Med..

[76] Kesim S., Oksuzoglu K., Ozguven S. (2023). Nocardia Infection with Adrenal Gland Abscess Mimicking Metastatic Lung Cancer on FDG PET/CT. Clin. Nucl. Med..

[77] Tejura N., Sonyey A. (2017). CMV-associated adrenal insufficiency in a renal transplant recipient. IDCases.

[78] Jain T.K., Karunanithi S., Bal C., Kumar R. (2017). 18F-FDG PET/CT Imaging in Adrenal Cryptococcosis. Clin. Nucl. Med..

[79] Cataño J., Porras J. (2020). Adrenal Paracoccidioidomycosis. Am. J. Trop. Med. Hyg..

[80] Sharma N., Ahlawat R.S., Singh H., Sharma C., Anuradha S. (2019). *Pneumocystis jirovecii* infection of bilateral adrenal glands in an immunocompetent adult: A case report. J. R. Coll. Physicians Edinb..

[81] Porntharukchareon T., Khahakaew S., Sriprasart T., Paitoonpong L., Snabboon T. (2019). Bilateral Adrenal Histoplasmosis. Balk. Med. J..

[82] Kwon Y.S., Lee H.W., Kim H.J. (2019). Paragonimus westermani infection manifesting as a pulmonary cavity and adrenal gland mass: A case report. J. Infect. Chemother..

[83] Sharma S.K., Tripathi M. (2020). Addison’s disease due to histoplasmosis of bilateral adrenal glands in a previously treated extrapulmonary tuberculosis case. Indian J. Med. Res..

[84] Langmaid T., Jassal K., Meher-Homji Z., Lee J.C., Serpell J., Yeung M., McMahon J., Grodski S. (2021). Disseminated nocardiosis with adrenal abscess masquerading as metastatic adrenal cancer in an immunocompetent adult. ANZ J. Surg..

[85] Jackson L.E., Shorman M. (2018). A Case of Bilateral *Nocardia francinia* Adrenal Abscesses in an Intravenous Drug-Using Splenectomized Patient with Tricuspid Endocarditis. Open Forum Infect. Dis..

[86] Pender M., Mehta N., Hamilton B.D., Swaminathan S. (2022). *Nocardia beijingensis* Isolated from an Adrenal Abscess in a Diabetic Host. Open Forum Infect. Dis..

[87] Yip S.W.Y., Li Y.L., Chu Y.L.E., Mak J.Y.H., Li J.Y.Y., Lee K.-H., Lee R. (2020). Adrenal and renal abscesses following glue embolization of gastric varices: A case description. Quant. Imaging Med. Surg..

[88] Rog C.J., Rosen D.G., Gannon F.H. (2016). Bilateral adrenal histoplasmosis in an immunocompetent man from Texas. Med. Mycol. Case Rep..

[89] Schrimashaw N. (1992). Effect of infection on nutritional status. Proc. Natl. Sci. Counc. B Life Sci..

[90] Du Bois E.F. (1938). The mechanism of Heat Loss and Temperature regulation. Ann. Intern. Med..

[91] Azzam I., Gilad S., Limor R. (2017). Ghrelin stimulation by hypothalamic-pituitary-adrenal axis activation depends on increasing cortisol levels. Endocr. Connect..

[92] Elshimy G., Chippa V., Jeong J.M. (2023). Adrenal Crisis. 2023 Feb 3. Stat Pearls (Internet).

[93] Sominsky L., Spencer S.J. (2014). Eating behavior and stress: A pathway to obesity. Front. Psychol..

[94] Erkut Z.A., Pool C., Swaab D.F. (1998). Glucocorticoids suppress corticotropin-releasing hormone in human hypothalamic neurons. J. Clin. Endocrinol. Metab..

[95] El-Radhi A.S. (2019). Pathogenesis of fever. Clinical Manual of Fever in Children.

[96] Jang W., Sohn Y., Park J.H., Pai H., Kim D.S., Kim B. (2021). Clinical Characteristics of Patients with Adrenal Insufficiency and Fever. J. Korean Med. Sci..

[97] Black S., Kushner I., Samols D. (2004). C-reactive Protein. J. Biol. Chem..

[98] Balli S., Shumway K.R., Sharan S. (2023). Physiology, Fever. 2022 Sep 11. StatPearls [Internet].

[99] Smith S. (2006). The Role of hypothalamic-pituitary-adrenal axis in neuroendocrine responses to stress. Dialogues Clin. Neurosci..

[100] Younes N., Bourdeau I., Lacroix A. (2021). Latent Adrenal Insufficiency: From Concept to Diagnosis. Front. Endocrinol..

[101] Rana M.V., Candido K.D., Raja O., Knezevic N.N. (2014). Celiac plexus block in the management of chronic abdominal pain. Curr. Pain Headache Rep..

[102] Megha R., Wehrle C.J., Kashyap S., Leslie S.W. (2023). Anatomy, Abdomen and Pelvis: Adrenal Glands (Suprarenal Glands) [Updated 2022 Oct 17]. StatPearls [Internet].

[103] Newhall D.A., Oliver R., Lugthart S. (2020). Anaemia: A disease or symptom?. Neth. J. Med..

[104] Sharma S., Nemeth E., Chen Y.H., Goodnough J., Huston A., Roodman G., Ganz T., Lichtenstein A. (2008). Involvement of hepcidin in the anemia of multiple myeloma. Clin. Cancer Res..

[105] De Mast Q., van Dongen-Leses E.C., Swinkles D.W., Nieman A.E., Roestenberg M., Druilhe P., Arens T.A., Luty A.J., Hermsen C.C., Sauerwein R.W. (2009). Mild increases in serum hepcidin and interleukin -6 concentrations impair iron incorporation in haemoglobin during an experimental human malaria infection. Br. J. Haematol..

[106] Miller C.B., Jones R.J., Piantados S., Abeloff M.D., Spivak J.L. (1990). Decreased erythropoietin response in patients with the anemia of cancer. N. Engl. J. Med..

[107] Mityling B.L., Singh J.A., Furne J.K., Ruddy J., Levitt M.D. (2006). Use of breath carbon monoxide measurements to access erythrocyte survival in subjects with chronic diseases. Am. J. Hematol..

[108] Gauer R., Forbes D., Boyer N. (2020). Sepsis: Diagnosis and Management. Am. Fam. Physician.

[109] Larkin C.M., Santos-Martinez M.J., Ryan T., Radomski M.W. (2016). Sepsis-associated thrombocytopenia. Thromb. Res..

[110] Franchini M., Veneri D., Lippi G. (2017). Thrombocytopenia and infections. Expert Rev. Hematol..

[111] Bedet A., Razazi K., Boissier F., Surenaud M., Hue S., Giraudier S., Brun-Buisson C., Mekontso Dessap A. (2018). Mechanisms of Thrombocytopenia During Septic Shock: A Multiplex Cluster Analysis of Endogenous Sepsis Mediators. Shock.

[112] Giustozzi M., Ehrlinder H., Bongiovanni D., Borovac J.A., Guerreiro R.A., Gąsecka A., Papakonstantinou P.E., Parker W.A. (2021). Coagulopathy and sepsis: Pathophysiology, clinical manifestations and treatment. Blood Rev..

[113] Tsirigotis P., Chondropoulos S., Frantzeskaki F., Stamouli M., Gkirkas K., Bartzeliotou A., Papanikolaou N., Atta M., Papassotiriou I., Dimitriadis G. (2016). Thrombocytopenia in critically ill patients with severe sepsis/septic shock: Prognostic value and association with a distinct serum cytokine profile. J. Crit. Care.

[114] Johansson D., Rasmussen M., Inghammar M. (2018). Thrombocytopenia in bacteraemia and association with bacterial species. Epidemiol. Infect..

[115] Oelkers W. (1996). Adrenal insufficiency. N. Engl. J. Med..

[116] Adrogué H.J., Tucker B.M., Madias N.E. (2022). Diagnosis and Management of Hyponatremia: A Review. JAMA.

[117] Liamis G., Milionis H.J., Elisaf M. (2011). Endocrine disorders: Causes of hyponatremia not to neglect. Ann. Med..

[118] Yoshida T., Masuyama H., Yamagata H., Miyabayashi M., Onishi S., Inaba Y., Takemoto M. (2022). The Incidence and Risk Factors of Hyponatremia in Pulmonary Tuberculosis. J. Endocr. Soc..

[119] Orth D.N. (1994). Adrenal insufficiency. Curr. Ther. Endocrinol. Metab..

[120] Napier C., Pearce S.H. (2012). Autoimmune Addison’s disease. Presse Med..

[121] Betterle C., Morlin L. (2011). Autoimmune Addison’s disease. Endocr. Dev..

[122] Grabarczyk M., Gorczyca M., Cieślik P., Hrycek A., Holecki M. (2023). Addison’s Disease in the Course of Recurrent Microangiopathic Antiphospholipid Syndrome—A Clinical Presentation and Review of the Literature. Medicina.

[123] Espinosa G., Cervera R., Font J., Asherson R.A. (2003). Adrenal involvement in the antiphospholipid syndrome. Lupus.

[124] Tallis P.H., Rushworth R.L., Torpy D.J., Falhammar H. (2019). Adrenal insufficiency due to bilateral adrenal metastases—A systematic review and meta-analysis. Heliyon.

[125] Alevritis E.M., Sarubbi F.A., Jordan R.M., Peiris A.N. (2003). Infectious causes of adrenal insufficiency. South Med. J..

[126] Paolo W.F., Nosanchuk J.D. (2006). Adrenal infections. Int. J. Infect. Dis..

[127] Arlt W., Allolio B. (2003). Adrenal insufficiency. Lancet.

[128] Araúz A.B., Papineni P. (2021). Histoplasmosis. Infect. Dis. Clin. N. Am..

[129] Maiga A.W., Deppen S., Scaffidi B.K., Baddley J., Aldrich M.C., Dittus R.S., Grogan E.L. (2018). Mapping Histoplasma capsulatum Exposure, United States. Emerg. Infect. Dis..

[130] Azar M.M., Loyd J.L., Relich R.F., Wheat L.J., Hage C.A. (2020). Current Concepts in the Epidemiology, Diagnosis, and Management of Histoplasmosis Syndromes. Semin. Respir. Crit. Care Med..

[131] Benedict K., Derado G., Mody R.K. (2016). Histoplasmosis-Associated Hospitalizations in the United States, 2001–2012. Open Forum Infect. Dis..

[132] Mallereau C.H., Todeschi J., Ganau M., Cebula H., Bozzi M.T., Romano A., Le Van T., Ollivier I., Zaed I., Spatola G. (2023). Pituitary Abscess: A Challenging Preoperative Diagnosis—A Multicenter Study. Medicina.

[133] Pearce E.N., Farwell A.P., Braverman L.E. (2003). Thyroiditis. N. Engl. J. Med..

[134] Lesh R., Hellums R., Pichardo P., Wong J., Pellitteri P., Purdy N., Stavrides K., Haugen T.W. (2023). Thyroid Abscess: A Case Series and Literature Review. Ear Nose Throat J..

[135] Yin D., Ji C., Zhang S., Wang J., Lu Z., Song X., Jiang H., Lau W.Y., Liu L. (2021). Clinical characteristics and management of 1572 patients with pyogenic liver abscess: A 12-year retrospective study. Liver Int..

[136] Singh A.K., Karmani S., Samanta J., Gupta P., Gupta V., Yadav T.D., Kumari S., Dutta U., Sinha S.K., Kochhar R. (2021). Splenic abscess in a tertiary care center in India: Clinical characteristics and prognostic factors. ANZ J. Surg..

[137] Schmidt P.N., Roug S., Hansen E.F., Knudsen J.D., Novovic S. (2014). Spectrum of microorganisms in infected walled-off pancreatic necrosis—Impact on organ failure and mortality. Pancreatology.

[138] Mowbray N.G., Ben-Ismaeil B., Hammoda M., Shingler G., Al-Sarireh B. (2018). The microbiology of infected pancreatic necrosis. Hepatobiliary Pancreat. Dis. Int..

[139] Rubilotta E., Balzarro M., Lacola V., Sarti A., Porcaro A., Artibani W. (2014). Current clinical management of renal and perinephric abscesses: A literature review. Urologia.

[140] Beishuizen A., Thijs L.G. (2003). Endotoxin and the hypothalamo-pituitary-adrenal (HPA) axis. J. Endotoxin. Res..

[141] Nacher M., Alsibai K.D. (2021). HIV associated Disseminated Histoplasmosis and Rare Adrenal Involvement: Evidence of Absence of Evidence. Front. Cell. Infect. Microbiol..

[142] Bhattacharya S., Kubiha S., Tyagi P., Feingold K.R., Anawalt B., Blackman M.R., Boyce A., Chrousos G., Corpas E., de Herder W.W., Dhatariya K., Dungan K., Hofland J. (2000). Fungi and Endocrine Dysfunction. (Updated 2021 Jun 25). Endotext (Internet).

[143] Frenkel J.K. (1962). Role of corticosteroids as predisposing factors in fungal diseases. Lab Invest. J. Tech. Methods Pathol..

[144] Vinnard C., Blumberg E.A. (2017). Endocrine and Metabolic Aspects of Tuberculosis. Microbiol. Spectr..

[145] Johnson P.T., Horton K.M., Fishman E.K. (2009). Adrenal mass imaging with multidetector CT: Pathologic conditions, pearls, and pitfalls. Radiographics.

[146] Papanicolas I., Woskie L.R., Jha A.K. (2018). Health Care Spending in the United States and Other High-Income Countries. JAMA.

[147] Osa S.R., Peterson R.E., Roberts R.B. (1981). Recovery of adrenal reserve following treatment of disseminated South American blastomycosis. Am. J. Med..

[148] Rana C., Kumari N., Krishnani N. (2011). Adrenal histoplasmosis: A diagnosis on fine needle aspiration cytology. Diagn. Cytopathol..

[149] Niemeyer C.S., Mescher T., Bubak A.N., Medina E.M., Hassell J.E., Nagel M.A. (2022). VZV Infection of Primary Human Adrenal Cortical Cells Produces a Proinflammatory Environment without Cell Death. Viruses.

